# Emerging biomarkers for improving pregnancy planning in multiple sclerosis

**DOI:** 10.3389/fneur.2024.1292296

**Published:** 2024-02-15

**Authors:** Juan Pablo Cuello, Ariana Meldaña Rivera, Enric Monreal, Ana Gómez Lozano, Ana Maria García Cano, Jose Manuel García Domínguez, José Ignacio Fernández Velasco, Lucienne Costa-Frossard França, Haydee Goicochea, Yolanda Higueras, Juan Antonio De León-Luis, Susana Sainz De La Maza, Noelia Villarrubia, Ignacio Arribas Gómez, Irene Ruiz Perez, Maria Luisa Martinez Ginés, Luisa María Villar

**Affiliations:** ^1^Department of Neurology, Hospital General Universitario Gregorio Marañón, Madrid, Spain; ^2^Health Research Institute Gregorio Marañón, Madrid, Spain; ^3^Department of Neurology, Hospital Universitario Ramón y Cajal, Universidad de Alcalá, Instituto Ramón y Cajal de Investigación Sanitaria (IRYCIS), Red Española de Esclerosis Múltiple (REEM), Red de Enfermedades Inflamatorias (REI), Madrid, Spain; ^4^Department of Clinical Biochemistry, Hospital Universitario Ramón y Cajal, Madrid, Spain; ^5^Department of Immunology, Hospital Universitario Ramón y Cajal, Universidad de Alcalá, Instituto Ramón y Cajal de Investigación Sanitaria (IRYCIS), Red Española de Esclerosis Múltiple (REEM), Red de Enfermedades Inflamatorias (REI), Madrid, Spain; ^6^Department of Public and Maternal and Child Health, School of Medicine, Complutense University of Madrid, Madrid, Spain; ^7^Department of Obstetrics and Gynecology, Hospital General Universitario Gregorio Marañón, Madrid, Spain

**Keywords:** Multiple sclerosis, pregnancy, neurofilament light chain, glial fibrillary acidic protein, antimüllerian hormone

## Abstract

**Background:**

Patient disability, relapse rate, and age are used for family planning in multiple sclerosis (MS). However, the need for more accurate biomarkers is widely recognized. We aimed to explore the influence of age on neurofilament light chain (sNfL), which reflects acute inflammation; glial fibrillary acidic protein (GFAP), associated with disability progression independent of relapses; and anti-Müllerian hormone (AMH), reflecting ovarian reserve, to provide a tailored family planning strategy.

**Methods:**

This case-control study included 95 MS patients and 61 healthy control women (HCW). sNfL and GFAP levels were measured using a sensitive single-molecule array assay. AMH levels were measured by the automated Elecsys^®^ Anti-Müllerian Hormone Assay.

**Results:**

We observed no significant differences in AMH values between MS patients and the control group within any of the age-matched categories. Age exhibited a negative correlation with AMH values in both groups, as expected. Nevertheless, our findings suggest a slight tendency toward reduced ovarian reserve in MS patients (rho MS patients = −0.67, *p* < 0.0001; rho HCW = −0.43, *p* = 0.0006). Interestingly, among the 76 MS participants under 40 years old, we identified ten individuals (13.1%) with AMH levels below 0.7 ng/ml, indicative of a low ovarian reserve, and an additional six individuals (7.8%) with AMH levels between 0.7 ng/ml and 0.9 ng/ml, suggesting a potential risk of premature ovarian failure. Conversely, sNfL and GFAP levels in the MS group exhibited high variability but showed no significant association with age intervals.

**Conclusion:**

We found no significant differences in AMH, sNfL or GFAP values between MS patients and the control group within any of the age-matched categories. The assessment of AMH, sNFL and GFAP levels at MS onset facilitates personalized therapeutic and family planning strategies for childbearing-age women.

## Graphical Abstract

A case-control study comprising 95 multiple sclerosis patients and 61 healthy control women. Evaluating Anti-Müllerian hormone, Neurofilament light-chain, and Glial Fibrillary Acidic Protein at disease onset enables personalized therapeutic and family planning strategies. 
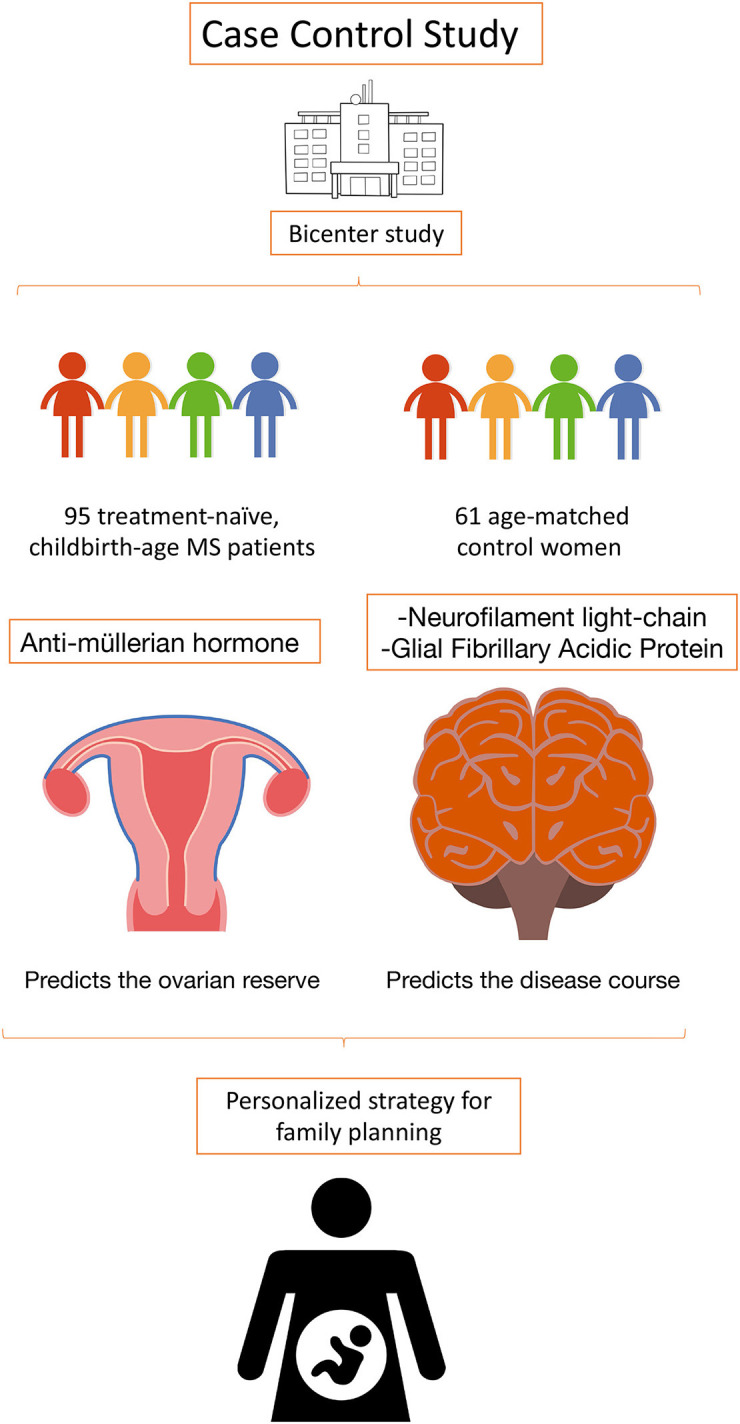

## Introduction

Multiple sclerosis (MS) is the most common degenerative neurologic disease in young adults, with an approximate female predominance of 3 to 1 (1). Early optimal treatment election proved to positively influence long-term disability outcomes (2). In MS, the treatment strategy is determined by different factors, such as relapse activity, MRI load burden at disease onset, and patient preference (2). Furthermore, in female patients who may consider becoming pregnant in the future, additional factors should be considered when choosing a disease-modifying treatment (DMT), such as pharmacodynamics and safety profile of the elected treatment, to reduce maternal and newborn morbidity (3, 4).

Pregnancy and puerperium are periods during which MS patients can experience changes in their inflammatory status, and there are limited biomarkers available with proven utility predicting short- or long-term MS activity during these phases (5, 6). Recently, serum neurofilament light chain (sNfL) has emerged as a biomarker of neuronal damage. Its levels have been associated with MS activity, disability scores, and treatment response, and their values at diagnosis can be used as a prognostic predictor (7, 8). Interestingly, sNfL can also be used as a surrogate biomarker of MS activity during pregnancy and puerperium (9). On the contrary, blood glial fibrillary acidic protein (GFAP) is a biomarker of astrocytic damage, and its levels correlate with clinical disability and with lesion burden in MS (10). It was proposed that assessing both sNfL and GFAP may be useful for identifying different stages of MS and for predicting prognosis and treatment response (11).

On the other hand, the anti-müllerian hormone (AMH) serum has been widely used in recent years as an index of ovarian functional potential, as its levels correlate with long-term fertility and age at menopause (12). AMH concentrations are relatively easy to measure, minimally invasive, and have good predictive value, making this test a valuable tool to evaluate ovarian reserve (13).

We aimed to explore differences in ovarian reserve, measured by AMH levels, between women with MS and healthy controls, and to evaluate the influence of age on NFL and GFAP levels in women with MS of childbearing age.

## Methods

### Participants

This case-control study was conducted at Hospital General Universitario Gregorio Marañón and Hospital Universitario Ramón y Cajal (Madrid, Spain) between January and December 2022. We included treatment naïve patients with MS at disease onset. MS diagnoses were made according to McDonalds criteria (14). As a control, we used age-matched healthy women. All participants were recruited consecutively from both tertiary hospitals. Patients and controls under 25 years old and above 45 were excluded from our study to match the mean childbearing-age in Western countries (15). Patients and controls were stratified by age, as normal values differ according to age ranges (12). As NfL and GFAP serve as biomarkers linked to neuronal damage and astrocytic activation, they were not assessed in the healthy control group. Finally, participants with known ovarian abnormalities or polycystic ovary syndrome were excluded.

### Serum samples

Serum samples of MS patients and healthy control women (HCW) were obtained from participants in a fasted state. After the collection, samples were aliquoted and stored at −80°C until analysis. Neurologists were blind to laboratory results during the study. sNfL and GFAP levels were measured with a sensitive single-molecule array. AMH levels were measured using the automated Elecsys^®^ anti-müllerian hormone assay (Roche Diagnostics International Ltd, Rotkreuz, Switzerland) according to the manufacturer's instructions (16, 17).

### Standard protocol approvals, registrations, and patient consents

The Ethics Committee of each center approved the study protocol. Written informed consent was obtained from all participating women.

### Statistical analyses

Descriptive analyses were performed. The results of the continuous variables are presented as the median and interquartile range (IQR) 25%−75%. Analyses were made using the Mann–Whitney U or chi-squared tests for categorical analysis. Missing items were imputed using mean imputation, and the correlation analysis was determined using Spearman's rho. For analysis purposes, we stratified participants by age to facilitate the study. All analyses were conducted with the STATA statistical package (College Station, TX, USA), and *p* < 0.05 were considered significant.

## Results

Initially, we consecutively recruited 100 MS patients and 65 HCW with childbearing potential. Some participants had to be excluded because of sample-handling issues. Finally, 95 MS patients and 61 HCW were analyzed. All MS patients had a relapsing form of the disease and were treatment naïve. Patients and control demographics are shown in Table 1.

**Table 1:** Demographics.

	**MS patients N: 95**	**Healthy control group N: 61**	**p**
Age (years) (25–75 IQR)	34.7 (29.7–40.5)	34 (30–41)	ns
Hormone contraception	No: 83 (87%) Yes 17 (13%)	No: 48 (78%) Yes 13 (22%)	ns
AMH < 0.7 ng/ml in patients on Hormone contraceptives	2 (2.1%)	4 (6.5%)	ns
Basal MRI T2 lesion load	1–3: 14 (14.7%) 2–9: 30 (31.6%) 10–50: 43 (45.3%) >50: 8 (8.4%)	NA	
Basal MRI T1 GAD+ lesion load	54 patients (54%) 2 (1–3)	NA	
Basal EDSS	1.5 (1–5)	NA	
IgG OCB	100 (100%)	NA	
LS IgM OCB	31 (30.5%)	NA	

MS, multiple sclerosis; IQR, interquartile range; AMH, Anti-müllerian hormone; MRI, Magnetic resonance imaging; OCB, Oligoclonal bands; IgG, immunoglobin G; LS OCB IgM, lipid-specific oligoclonal IgM bands. ns, non-significant, meaning p:> 0.05.

We explored the potential bias that hormonal contraception could have on AMH values (Table 1). Seventeen MS patients were on hormonal contraception during the study, but only two (18%) had AMH values below 0.7 ng/ml (patients aged 39 and 41 years). In the control group, 13 (22%) participants were on hormonal contraception, and four (30%) had AMH values below 0.7 ng/ml (HCW aged 30, 38, 42, and 42).

**Figure 1:**
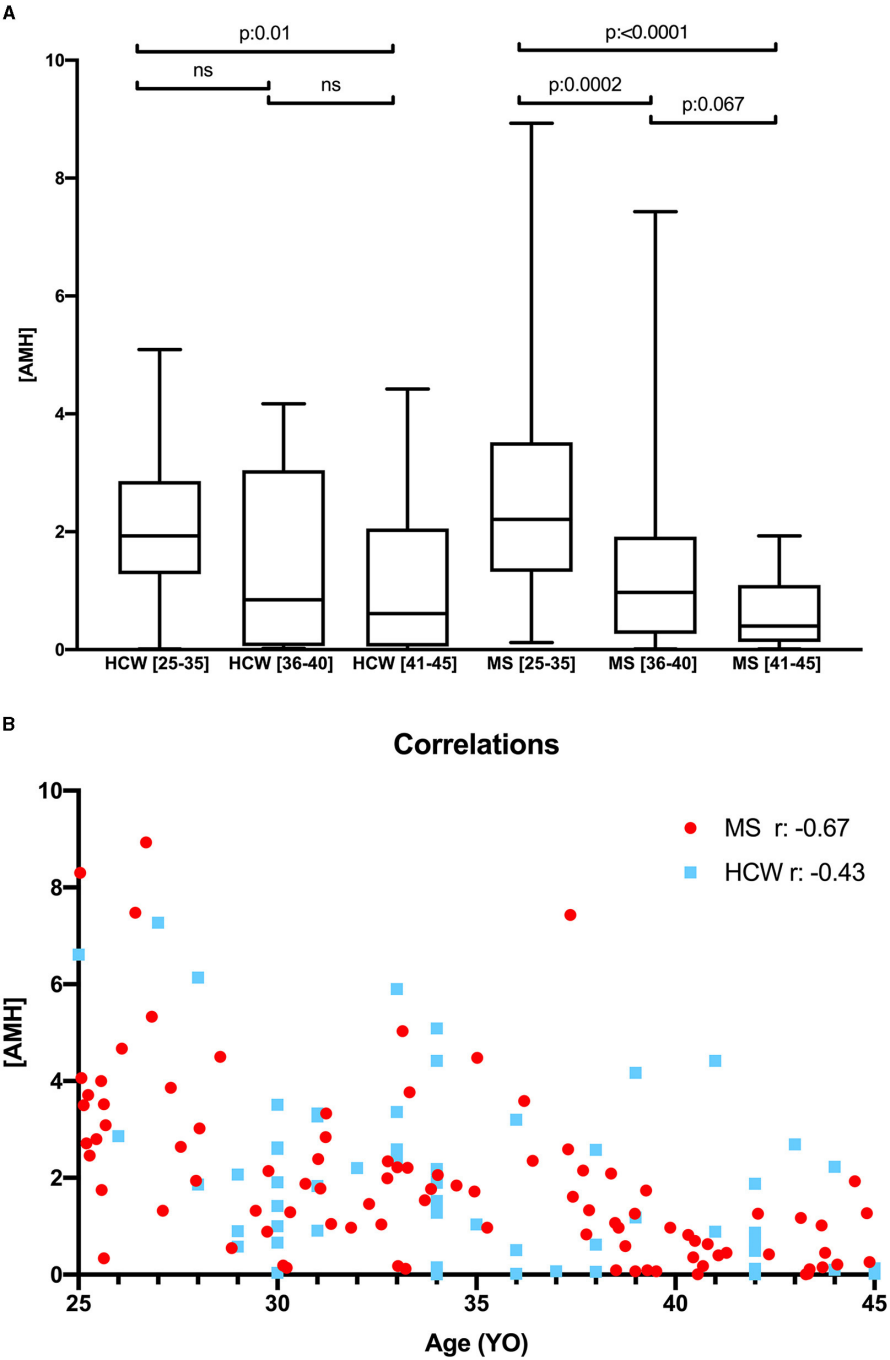
**(A)** Anti-müllerian *hormone* levels in MS patients and HCW, according to age stratification; **(B)** Spearman correlation test between AMH and age MS patients and HCW. MS, multiple sclerosis; AMH, anti-müllerian *hormone; r*, Spearman's rho; YO, years old.

Next, we evaluated the effect of age. It negatively correlated with AMH values in patients and controls. We found no difference in AMH values between MS patients and controls in any age-matched group (Figure 1A). However, we found a significant decrease among MS groups, a trend not observed in the control group. This is probably due to a greater variability in the values observed in MS group for ages younger than 41. This suggests that MS patients could have a tendency toward a lower ovarian reserve (rho MS patients−0.67, *p*: < 0, 0001; rho HCW−0.43, p: 0.0006) (Figure 1B). Along this line, 27 (29%) of MS patients had AMH concentrations below 0.7 ng/ml, of which 17 (60%) were in the 40- to 45-year-old group, four (14.8%) in the 36–40 group, and six (22.2%) were below 35 years old. More interestingly, six out of seven MS patients with AMH values between 0.7 ng/ml and 0.9 ng/ml were under the age of 40. Notably, this predominance was not observed in healthy women (Table 2).

**Table 2:** Participants with low ovarian reserve.

**AMH < 0.7 ng/ml**	**MS: 27**	**HCW:** **14**	***p***
25–35	6 (22.2%)	3 (21.5%)	ns
36–40	4 (14.8%)	4 (28.5%)	ns
41–45	17 (63%)	7 (50%)	ns
**AMH 0.7–0.9 ng/ml**	**7**	**5**	
25–35	1 (14.2%)	1 (20%)	ns
36–40	5 (71.6%)	1 (20%)	ns
41–45	1 (14.2%)	3 (60%)	ns

MS, multiple sclerosis; HCW, healthy control women; AMH, anti-müllerian hormone; ns, non-significant, meaning p:> 0.05.

**Figure 2:**
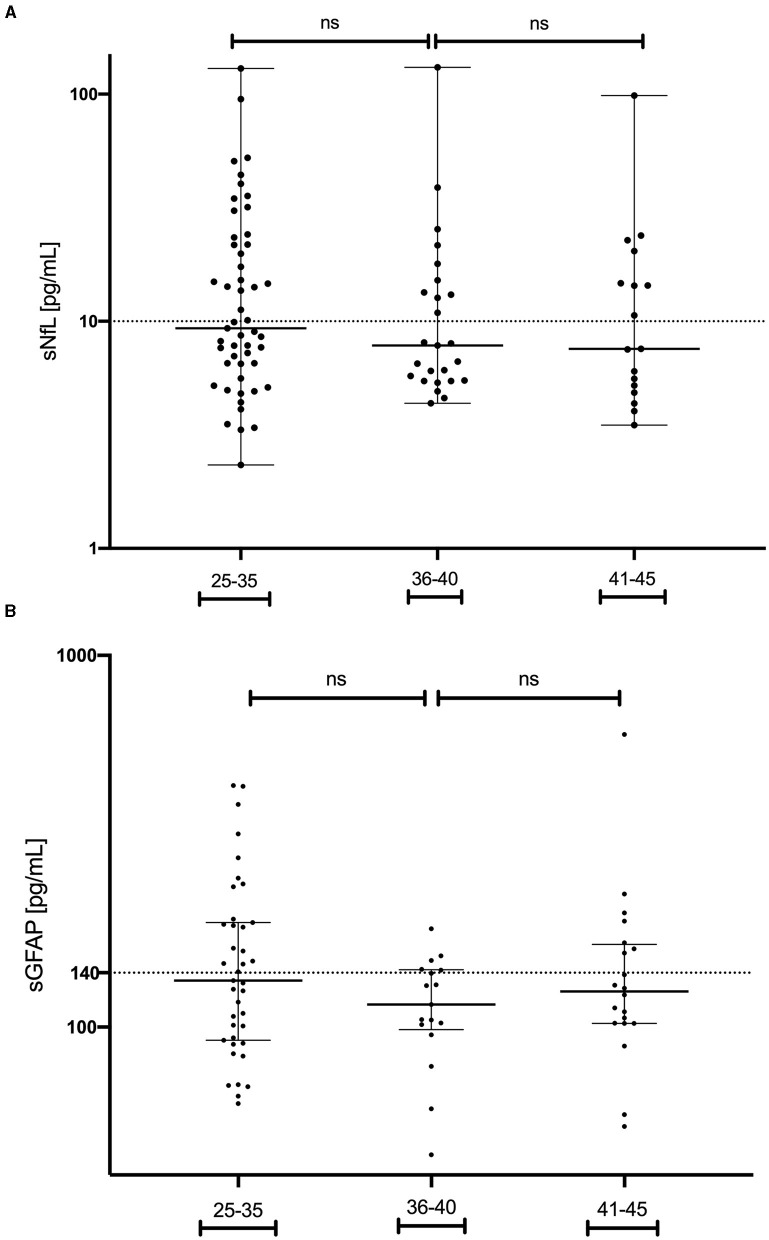
Serum neurofilament light chain levels in MS patients **(A)** and glial fibrillary acidic protein levels **(B)** in MS patients, categorized by age groups. MS, multiple sclerosis; GFAP, glial fibrillary acidic protein; sNfL, serum neurofilament light chain; ns, non-significant.

Finally, we evaluated both sNfL and GFAP values in the MS group and found that the stratification by age did not influence their concentrations, with a normal (Gaussian) distribution in each group of patients (Figure 2).

## Discussion

Family planning encompasses various factors, including but not limited to patient education, contraceptive accessibility, hospital protocols, and government policies. These elements aid individuals in making informed decisions about the timing of pregnancy, thereby reducing the incidence of unintended pregnancies. Consequently, Western countries have witnessed a rise in maternal age during pregnancy in recent decades, with negative repercussions on conception rates and the health of both mothers and offspring. Generally, women undergo a progressive decline in fecundity as they pass through their reproductive years, and this is related to different causes, such as detrimental oocyte quality, ovulation efficiency, sexual function, and uterine health (18). In addition, advanced maternal age can be associated with potential adverse outcomes that may include stillbirth, miscarriage, ectopic pregnancy, multiple births, congenital malformations, and more medical interventions at birth, especially in patients over 40 years old (19). In our study, we assessed how age stratification can impact the levels of AMH, GFAP, and sNfL at MS onset in women of childbearing age. Knowing both ovarian reserve and the MS evolution could facilitate the development of personalized treatment strategies and family planning.

In family planning, screening for ovarian reserve is a method used for predicting a woman's probability to achieve pregnancy in the future (20). The ovarian reserve can be estimated by calculating the ovarian antral follicles using ultrasound imaging or by using different biochemical methods. Recently, serum AMH has become one of the most commonly used techniques for testing ovarian reserve, as it has shown a good correlation with the transvaginal ultrasound counting of the antral follicles count (21). Interestingly, AMH levels in peripheral blood remain stable throughout menstrual cycles, allowing a convenient assessment at any time. The primary role of AMH in the adult ovary is to limit the formation of primary follicles by inhibiting excessive follicle recruitment by using follicle-stimulating hormone.

In the clinical field, AMH values above 1.77 ng/mL are considered indicative of an optimal ovarian reserve as they correlate positively with the optimal quantity of follicle reserve in the ovary. This cutoff has positive implications both for spontaneous and *in vitro* fecundation (22). By contrast, AMH levels below 0.7 ng/mL are associated with reduced fecundability both by natural intercourse or assisted insemination (23). Levels between 0.7 ng/mL and 0.9 ng/mL may help to identify women with a low ovarian reserve and are approaching menopause. As a limitation, contraceptive hormone therapy could be associated with a diminished AMH concentration, reversible once it ends (24). In our research, we found similar percentages of women on contraceptive therapy between patients and controls, with only 2 (2.1%) MS patients having levels below 0.7 ng/ml. Those two MS patients were 39 and 41 years old, so their AMH levels can be considered normal according to age. In our study, AMH levels diminished with participants' age as expected. There was no significant difference between MS patients and controls, consistent with recently published data, supporting the notion that MS does not impact fertility (25). However, we found a tendency of having lower values in young MS patients. In fact, 10 of 76 MS patients under the age of 40, matching the age at which MS patients try to get pregnant in our population area (26), had AMH values < 0.7 ng/ml. Six additional patients in their thirties had AMH values between 0.7 ng/ml and 0.9 ng/ml indicating diminished ovarian reserve, and therefore, compromised fertility.

Aging has been identified as a factor correlating with the longitudinal trajectories of sNfL and GFAP (27, 28). However, in our study, the values of sNfL and GFAP at MS onset did not exhibit a correlation with age stratification among our patients. This lack of correlation could be attributed to the specific focus of our research on young participants in their childbearing years. Nevertheless, given that sNfL and GFAP are associated with inflammation status and disability worsening in MS, we believe that both variables should be considered in family planning at disease onset (8, 29).

Our study suggests that evaluating serum levels of AMH, sNfL, and GFAP at MS onset enables the identification of individuals with diminished ovarian reserve and assesses disease activity, severity, and potential treatment response (8). Having this information at the disease onset allows clinicians to identify individuals who may benefit from an early, high-efficacy treatment strategy, thereby potentially delaying pregnancy. Conversely, in cases where ovarian reserve is compromised, clinicians must discuss the advantages of prioritizing pregnancy or even consider the option of oocyte cryopreservation to postpone gestation. As a result, patients and healthcare providers would be able to personalize the treatment strategy and make informed decisions regarding family planning. In Table 3, we suggest the different factors that should be considered to facilitate a personalized strategy for family planning in MS.

**Table 3:** Clinical and fine-tuning approaches for optimal family planning in MS.

	**Variable**	**Optimal situation**	**Suboptimal situation**
Clinical	Age	Younger age	Older age
Fine-tuning	AMH	>1.77 ng/ml	< 0.7 ng/ml
Clinical	AAR before conception	0	≥1
Fine-tuning	sNfL at conception	< 10 pg/ml	>10 pg/ml
Clinical	EDSS before conception	< 3	≥ 3
Fine-tuning	GFAP at conception^*^	< 140 pg/mL	≥140 pg/mL

Clinical and laboratory factors recommended for preconception planning in MS. The reference values for AMH, sNfL, and GFAP used in this table were described in previous publications (8, 11, 22). ARR, annual relapse rate; AMH, anti-müllerian hormone; MS, multiple sclerosis, sNfL, Serum neurofilament light chain; EDSS, Expanded Disability Status Scale; GFAP, Glial fibrillary acidic protein; ^*^: reference values for patients aged below 40 years old.

## Conclusion

The best time to become pregnant in MS should be autonomously decided by any individual, after being informed by their healthcare providers. Family planning should be addressed at the disease onset in childbearing-age women, even if there is no immediate pregnancy desire, because disease prognostic and pregnancy-related safety issues could influence treatment election. No differences were detected in the ovarian reserve, measured by AMH levels, between women of childbearing age in MS patients and controls. Additionally, no differences in GFAP and sNfL levels were found among the different age groups of women with MS in the childbearing age range.

## Data availability statement

The raw data supporting the conclusions of this article will be made available by the authors, without undue reservation.

## Ethics statement

The studies involving humans were approved by the Hospital General Universitario Gregorio Marañon and Hospital General Ramón y Cajal. The studies were conducted in accordance with the local legislation and institutional requirements. The participants provided their written informed consent to participate in this study.

## Author contributions

JC: Conceptualization, Data curation, Investigation, Methodology, Resources, Validation, Visualization, Writing—original draft, Writing—review & editing, Formal analysis, Project administration. AM: Conceptualization, Data curation, Investigation, Methodology, Writing—review & editing. EM: Data curation, Methodology, Writing—review & editing. AG: Data curation, Methodology, Writing—review & editing. AMG: Data curation, Methodology, Writing—review & editing. JG: Methodology, Writing—review & editing. JF: Data curation, Methodology, Writing—review & editing. LC-FF: Data curation, Methodology, Writing—review & editing. HG: Data curation, Methodology, Writing—review & editing. YH: Data curation, Methodology, Writing—review & editing. JL-L: Data curation, Methodology, Writing—review & editing. SS: Data curation, Methodology, Writing—review & editing. NV: Methodology, Writing—review & editing. IA: Methodology, Writing—review & editing. IR: Methodology, Writing—review & editing. MM: Conceptualization, Data curation, Methodology, Validation, Writing—review & editing. LV: Conceptualization, Formal analysis, Investigation, Methodology, Resources, Software, Validation, Visualization, Writing—original draft, Writing—review & editing.
